# Antitoxin autoregulation of *M. tuberculosis* toxin-antitoxin expression through negative cooperativity arising from multiple inverted repeat sequences

**DOI:** 10.1042/BCJ20200368

**Published:** 2020-06-26

**Authors:** Izaak N. Beck, Ben Usher, Hannah G. Hampton, Peter C. Fineran, Tim R. Blower

**Affiliations:** 1 Department of Biosciences, Durham University, Stockton Road, Durham DH1 3LE, U.K.; 2 Department of Microbiology and Immunology, University of Otago, PO Box 56, Dunedin 9054, New Zealand

**Keywords:** co-operativity, *Mycobacterium tuberculosis*, regulation, structural biology, toxin-antitoxin system, transcription

## Abstract

Toxin-antitoxin systems play key roles in bacterial adaptation, including protection from antibiotic assault and infection by bacteriophages. The type IV toxin-antitoxin system AbiE encodes a DUF1814 nucleotidyltransferase-like toxin, and a two-domain antitoxin. In *Streptococcus agalactiae*, the antitoxin AbiEi negatively autoregulates *abiE* expression through positively co-operative binding to inverted repeats within the promoter. The human pathogen *Mycobacterium tuberculosis* encodes four DUF1814 putative toxins, two of which have antitoxins homologous to AbiEi. One such *M. tuberculosis* antitoxin, named Rv2827c, is required for growth and whilst the structure has previously been solved, the mode of regulation is unknown. To complete the gaps in our understanding, we first solved the structure of *S. agalactiae* AbiEi to 1.83 Å resolution for comparison with *M. tuberculosis* Rv2827c. AbiEi contains an N-terminal DNA binding domain and C-terminal antitoxicity domain, with bilateral faces of opposing charge. The overall AbiEi fold is similar to Rv2827c, though smaller, and with a 65° difference in C-terminal domain orientation. We further demonstrate that, like AbiEi, Rv2827c can autoregulate toxin-antitoxin operon expression. In contrast with AbiEi, the P*_rv2827c_* promoter contains two sets of inverted repeats, which bind Rv2827c with differing affinities depending on the sequence consensus. Surprisingly, Rv2827c bound with negative co-operativity to the full P*_rv2827c_* promoter, demonstrating an unexpectedly complex form of transcriptional regulation.

## Introduction

Toxin-antitoxin (TA) systems are encoded by genetic loci that are widely distributed throughout prokaryotic genomes. They can play pivotal roles in bacterial physiology and in managing stress responses, helping bacteria to survive nutrient limitation, immune system attack, antibiotic treatment and predation by bacteriophages [1–5]. TA systems are commonly found on mobile genetic elements, contributing to the stability of plasmids, superintegrons, cryptic prophages and conjugative transposons [6–8]. The majority of TA systems encode two components, a toxic protein that generally targets essential cellular processes, and an antagonistic antitoxin [4]. This antitoxin negates toxin activity when cells are growing in favorable conditions. Under stressful conditions, the antitoxin is preferentially degraded and the toxin is released, thereby reducing growth rate as a means to survive with minimal metabolic burden until favorable conditions return [9,10]. Activation of the toxin following bacteriophage infection can also lead to the removal of the infectious bacteriophage particle from the environment, thereby providing a population level protection from viruses referred to as abortive infection (Abi) [11,12].

**Figure 1.**
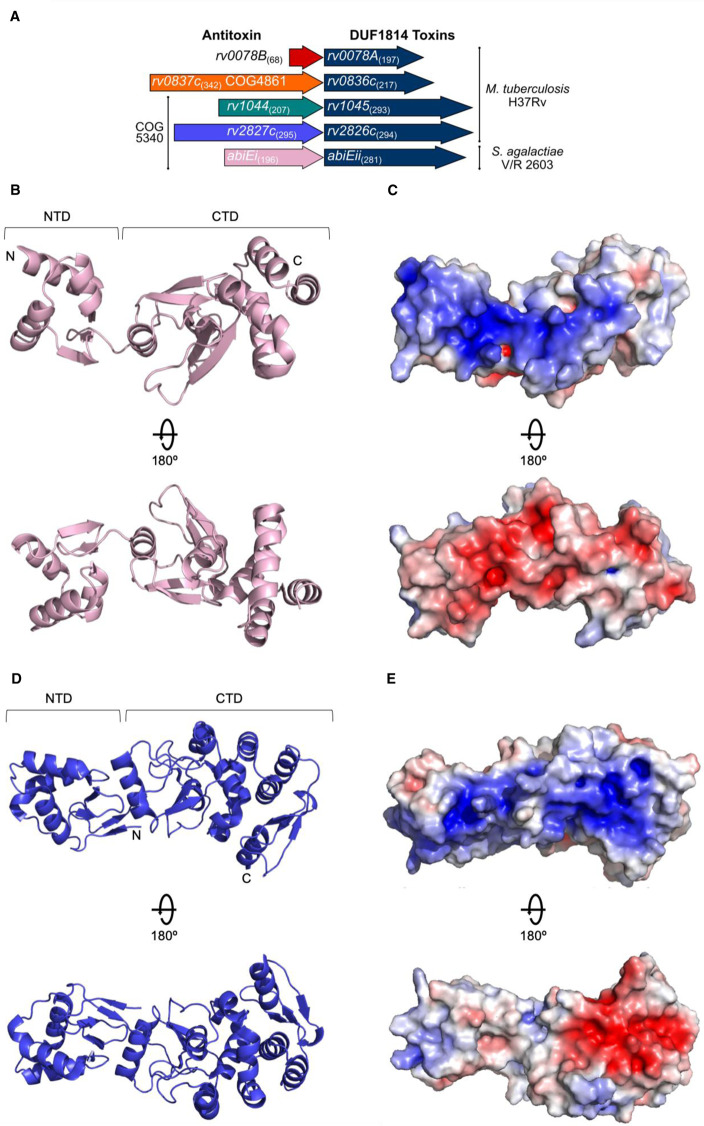
Antitoxin AbiEi is a two-domain protein with bilateral opposingly charged faces. (**A**) Scaled representation of the four *M. tuberculosis* TA systems containing NTase-like toxin genes and AbiE from *S. agalactiae*. Numbers in parentheses indicate amino acid length. All five toxins are DUF1814 proteins; Rv1044, Rv2827c and AbiEi are COG5340-containing antitoxins. Putative antitoxin Rv0837c is a COG4861 protein and the significantly shorter putative antitoxin Rv0078B is unclassified. The four *M. tuberculosis* systems were re-named as shown. (**B**) AbiEi antitoxin structure shown in pink cartoon representation, in two views rotated 180°. (**C**) Electrostatic potential of AbiEi, posed as per (**B**), with electropositive charge in blue and electronegative charge in red. (**D**) Previously solved Rv2827c structure shown in blue cartoon representation, in two views rotated 180° (PDB: 1ZEL). (**E**) Electrostatic potential of Rv2827c, posed as per (**D**), colored as per (**C**).

TA systems have been divided into six types according to the nature of the toxin and antitoxin (whether they are RNA or protein), and the mechanism of toxin antagonism [4]. Type IV systems differ from all others in that the antitoxin and toxin do not directly interact, instead, the antitoxin antagonizes the activity of the toxin [13–15]. There are multiple examples wherein TA systems provide a phage-resistant Abi phenotype, although not all identified Abi systems act as bona fide TA systems [5,15–20]. A recently characterized Abi system, AbiE from *Streptococcus agalactiae* V/R 2603, has been shown to act as a type IV TA system [15]. AbiE encodes a DUF1814-family toxin (AbiEii), and a COG5340-family antitoxin (AbiEi) (Figure 1A) [15]. The *S. agalactiae* AbiE COG5340 antitoxin will herein be referred to as AbiEi. AbiEii is a putative nucleotidyltransferase (NTase) that specifically binds GTP [15]. This DUF1814 family is widespread, present in over 5500 bacterial, archaeal and fungal genomes, though not all examples are genetically linked to putative antitoxins.

TA systems are remarkably abundant in *Mycobacterium tuberculosis*, which encodes more than 80 examples, and these are thought to have contributed to *M. tuberculosis* having become the most successful human pathogen [21–24]. *M. tuberculosis* H37Rv encodes four DUF1814-family NTase-like putative toxins, namely Rv0078A, Rv0836c, Rv1045 and Rv2826c (Figure 1A). Akin to AbiEii from *S. agalactiae*, both Rv1045 and Rv2826c have a cognate COG5340-family antitoxin (Figure 1A). Transposon mutagenesis studies have identified the cognate antitoxins of these systems (Rv1044 and Rv2827c) as essential for laboratory growth [25,26], suggesting that Rv1045 and Rv2826c toxins are functional in *M. tuberculosis*. The *M. tuberculosis* COG5340 proteins will herein be referred to by their respective ‘Rv’ identifiers, Rv1044 and Rv2827c. Characterizing and understanding the regulation of these loci is of interest for developing new therapies against the pathogen.

Autoregulation of TA system expression is a hallmark of type II TA systems and can be either positive or negative [27,28]. The antitoxin AbiEi from *S. agalactiae* has been biochemically characterized [15,29] and functions as both an antitoxin and a transcriptional repressor. That is, AbiEi negatively autoregulates *abiE* expression. Here, the gene product suppresses its own production, through positively co-operative binding of two AbiEi monomers to inverted repeats in the promoter region. Full length AbiEi is required for negative autoregulation and induced bending of the promoter DNA. We previously proposed that this bending was facilitated by the two AbiEi monomers interacting via their C-terminal domains (CTDs) [29]. In contrast with type II autoregulation, for which conditional co-operativity is observed, co-expression of the cognate toxin AbiEii does not enhance transcriptional repression [15]. We therefore sought to determine the similarities in the structure and function of AbiEi and Rv2827c. While the structure of the *M. tuberculosis* putative antitoxin Rv2827c has been solved as part of a structural genomics initiative [30], its biological function was not explored and it has not been biochemically characterized. We present the solved structure of *S. agalactiae* AbiEi, demonstrating structural homology between the COG5340 antitoxins, and biochemically characterize the molecular interactions underpinning transcriptional repression by Rv2827c. Interestingly, this is a more complex autoregulatory system than previously seen for AbiEi [29].

## Materials and methods

### Bacterial strains and culture conditions

*E. coli* DH5α (Invitrogen), BL21 (DE3) (Invitrogen) and ER2566 (New England Biolabs) were routinely grown at 37°C in Luria-Broth (LB), M9 minimal (M9M), or 2× YT media supplemented when necessary with ampicillin (Ap, 50 µg/ml), spectinomycin (Sp, 100 µg/ml), tetracycline (Tc, 10 µg/ml), isopropyl-β-D-thiogalactopyranoside (IPTG, 1 mM), L-arabinose (L-ara, 0.1% w/v) or D-glucose (glu, 0.2% w/v). Bacterial cell density was measured using a WPA Biowave C08000 at 600 nm (OD_600_).

### DNA isolation and manipulation

All oligonucleotides used in this study were obtained from Integrated DNA Technologies (Supplementary Table S1). Plasmid and PCR-amplified DNAs were purified using Monarch kits (NEB). Digests, ligations, transformations and agarose gel electrophoresis steps were performed by standard techniques. All constructed plasmids (Supplementary Table S2) were confirmed by sequencing using an ABI 3730 DNA sequencer and 4Peaks.

Protein expression constructs were made by Ligation Independent Cloning (LIC) [31]. Target genes were cloned into plasmid pSAT1-LIC, which generates N-terminal His_6_-SUMO fusions with the target ORF. Primers TRB1048/TRB1049 were used to amplify *abiEi* from pRLD30, for LIC insertion into pSAT1-LIC, producing pTRB525. Primers TRB1022/TRB1023 were used to amplify *rv2827c* from pPF658, also for LIC insertion into pSAT1-LIC, producing pTRB493. Primers TRB1018/TRB1019 were used to amplify *rv1044* from *M. tuberculosis* H37Rv genomic DNA (ATCC), again for LIC insertion into pSAT1-LIC, producing pTRB491.

For promoter activity assays, regions upstream of *abiEi*, *rv2827c* and *rv1044* were cloned into pRW50 [32]. The 99 bp region upstream of *abiEi* was amplified from pPF680 using primers TRB1072/TRB1047, then digested with EcoRI/HindIII and ligated into pRW50 cut with the same enzymes, producing pTRB486. The 500 bp regions upstream of *rv2827c* and *rv1044* were amplified from H37Rv genomic DNA, using primers TRB1042/TRB1043 and TRB1040/TRB1041, respectively. The amplicons were digested with EcoRI/HindIII and ligated into pRW50 cut with the same enzymes, producing pTRB484 and pTRB483, respectively. Antitoxin genes *abiEi, rv2827c and rv1044* were cloned into pTA100, a pQE-80 derivative [5]. *S. agalactiae abiEi* was amplified from pRLD30 using primers TRB1052/TRB1053, then digested with EcoRI/HindIII and ligated into pTA100 cut with the same enzymes, producing pTRB481. *M. tuberculosis rv2827c* and *rv1044* were amplified from H37Rv genomic DNA, using primers PF1334/PF1335 and PF1330/PF1331, respectively. The amplicons were digested with NdeI/SpeI and ligated into pTA100 cut with the same enzymes, producing pPF658 and pPF658, respectively.

### Protein expression and purification

To express AbiEi, Rv2827c and Rv1044 for crystallization and/or biochemistry, *E*. *coli* ER2566 (for native protein) or BL21 (DE3) (for labeled protein) were transformed with pTRB525, pTRB493 or pTRB491, respectively. For native protein, overnight cultures were re-seeded 1 : 100 into 2 L flasks containing 1 L 2× YT. Cells were grown at 150 rpm in 37°C until an OD_600_ of 0.6–0.8 was reached, whereupon expression was induced by the addition of IPTG (1 mM). Cells were left to grow for 16 h at 17°C, shaking at 150 rpm.

For incorporation of selenomethionine into AbiEi, the SeMet kit (Molecular Dimensions) was used. Starter cultures of BL21 (DE3) pTRB525, starter cultures were grown for 8 hours in LB at 37 °C with 200 rpm shaking. This culture was used to inoculate (1 : 500) a 50 ml overnight of Molecular Dimensions Selenomethionine Base medium supplemented with Molecular Dimensions Nutrient Mix. This overnight was then used to inoculate (1 : 100) 1 L of the same Base Medium with Nutrient Mix and cells were grown at 37°C with 180 rpm shaking. At OD_600_ 0.8, cells were pelleted by centrifugation at 4200×***g***, resuspended in fresh Base Medium with Nutrient Mix (Molecular Dimensions) and supplemented with an amino acid mix to promote feedback inhibition of methionine synthesis (0.1 mg/ml L-lysine hydrate, 0.1 mg/ml L-threonine, 0.1 mg/ml L-phenylalanine, 0.05 mg/ml L-leucine, 0.05 mg/ml L-isoleucine, 0.05 mg/ml L-valine). Cells were grown for a further 30 min at 37°C with shaking at 180 rpm before the addition of 250× SelenoMethionine Solution (Molecular Dimensions) to a final concentration of 40 µg/ml. Cells were grown for a further 15 min at 37°C with shaking at 180 rpm before antitoxin expression was induced with IPTG (1 mM), and samples were left to grow overnight at 175 rpm in 18°C.

For native protein purification, bacteria were harvested by centrifugation at 4200×***g*** and the pellets were resuspended in buffer A500 (20 mM Tris–HCl pH 7.9, 500 mM NaCl, 5 mM imidazole and 10% glycerol). Cells were lysed by sonication at 40 kpsi, then centrifuged (45 000×***g***, 4°C). The clarified lysate was next passed over a HisTrap HP column (GE Healthcare), washed for ten column volumes with A500, followed by 10 column volumes of buffer A100 (20 mM Tris–HCl pH 7.9, 100 mM NaCl, 5 mM imidazole and 10% w/v glycerol), then eluted directly onto a HiTrap Q HP column (GE Healthcare) with buffer B100 (20 mM Tris–HCl pH 7.9, 100 mM NaCl, 250 mM imidazole and 10% w/v glycerol). The Q HP column was transferred to an Äkta Pure (GE Healthcare), washed with three column volumes of A100, then proteins were eluted using a gradient from 100% A100 to 100% buffer C1000 (20 mM Tris–HCl pH 7.9, 1000 mM NaCl and 10% w/v glycerol). Fractions containing the protein peak were analyzed by SDS–PAGE, pooled and incubated overnight at 4°C with hSENP2 SUMO protease (SENP) to cleave the His_6_-SUMO tag from the target protein. The following day, the samples were passed through a second HisTrap HP column and the flow-through fractions containing untagged target protein were collected. The same procedure was used for labeled protein, except seleno-AbiEi precipitated on column at A100, so contaminants were removed with B100, and remaining folded seleno-AbiEi was eluted with B500, followed by SENP cleavage and a second HisTrap column purification. Proteins were dialyzed overnight at 4°C into buffer X (20 mM Tris–HCl pH 7.9, 200 mM NaCl and 2.5 mM DTT). Crystallization samples were concentrated, quantified and stored on ice, then either used immediately or flash-frozen in liquid N_2_ for storage at −80°C.

### Protein crystallization

Native and selenomethionine-derivatized AbiEi were concentrated to 12 mg/ml in buffer X (see above). Initial native AbiEi crystallization screens were performed using commercial screens (Molecular Dimensions) set with an Innovadyne Screenmaker robot, making 200 : 100 nl and 100 : 100 nl protein:condition sitting drops at 18°C. After initial screening and optimization, native AbiEi formed thick needles in 0.02 M Sodium/Potassium phosphate, 0.1 M Bis-Tris Propane pH 6.5, 20% PEG 3350. Selenomethionine-derivatized AbiEi crystals grew in 0.2 M Sodium acetate trihydrate, 0.1 M Bis-Tris Propane pH 6.5, 15% PEG 3350. To harvest, 20 µl of condition reservoir was added to 20 µl of glycerol and mixed quickly by vortexing; an equal volume of this mixture was then added to the drop volume. After addition of cryo buffer, crystals were immediately extracted using a nylon loop and flash-frozen in liquid N_2_.

### X-ray data collection and structure determination

Diffraction data were collected at Diamond Light Source on beamline I03 (AbiEi native and AbiEi selenomethionine-derivatized) (Table 1). Single, 360°, datasets were collected from three native AbiEi crystals and merged using iSpyB (Diamond Light Source). Two, 360°, datasets from AbiEi selenomethionine-derivatized crystals measured at the selenium peak (0.9793 Å) were also merged using iSpyB. An additional AbiEi selenomethionine-derivatized dataset was collected at selenium high remote (0.9641 Å) wavelength. Diffraction data were processed with XDS [33,34], and then AIMLESS from CCP4 [35] was used to corroborate the spacegroups (Table 1). The crystal structure of AbiEi was solved by MAD, by providing the SHELX suite [36] in CCP4 with the native, peak and high remote datasets. The solved starting model for AbiEi was built in REFMAC [37] and BUCCANEER [38]. The model was then iteratively refined and built using PHENIX [39] and COOT [40], respectively. The quality of the final model was assessed using COOT and the wwPDB validation server [41]. Structural figures were generated using PyMol (Schrödinger). Structural alignments were performed using PROMALS3D [42].

**Table 1:** Crystallographic data collection and refinement statistics

	AbiEi Native	AbiEi Se-Peak	AbiEi Se-High Remote
PDB ID Code	6Y8Q	-	-
Number of crystals	3	2	1
Beamline	Diamond I03	Diamond I03	Diamond I03
Wavelength, Å	0.9763	0.9793	0.9641
Resolution range, Å	42.11–1.83 (1.86–1.83)^a^	42.58–2.14 (2.19–2.14)	53.57–2.17 (2.23–2.17)
Space group	P1	P1	P1
Unit cell, *a b c* (Å), *α β γ* (°)	34.24 80.85 122.17, 102.48 96.74 100.47	34.78 81.37 122.99, 101.72 97.18 101.16	34.85 81.38 123.00, 101.74 97.31 101.19
Total reflections	207 238(10 275)	443 813(13 873)	129 874(8557)
Unique reflections	106 620(5213)	69 714(4469)	65 917(4312)
Multiplicity	1.9	6.4	2.0
Completeness (%)	97.4 (96.1)	99.0 (97.1)	97.9 (91.9)
Mean I/sigma(I)	7.6	6.9	6.1
*R*_merge_	0.038 (0.691)	0.169 (1.036)	0.080 (0.593)
*R*_meas_	0.053 (0.977)	0.184 (1.260)	0.113 (0.839)
CC_1/2_	0.999 (0.471)	0.991 (0.463)	0.992 (0.599)
*R*_work_	0.1812 (0.2812)	-	-
*R*_free_	0.2092 (0.3100)	-	-
No. of non-hydrogen			
atoms	7116	-	-
Macromolecules	6397	-	-
Ligands	62	-	-
Solvent	657	-	-
Protein Residues	769	-	-
RMSD (bonds, Å)	0.012	-	-
RMSD (angles, °)	1.32	-	-
Ramachandran			
favored (%)	98.68	-	-
Ramachandran			
allowed (%)	1.32	-	-
Ramachandran			
outliers (%)	0.00	-	-
Average B-factor	39.61	-	-
Macromolecules	39.04	-	-
Ligands	46.01	-	-
Solvent	44.60	-	-

### Electrophoretic mobility shift assays

Conservation of IR sequences was determined using MView [43] and WebLogo [44]. Promoter region probes were amplified from synthesized templates (Supplementary Table S1). Each template was made with a common downstream region, matching the initial part of *lacZ* from pRW50. For each probe template, a unique upstream forward primer was used in combination with a common reverse primer, which was either untagged or had been conjugated to a fluorescein tag for visualization (Supplementary Table S1). The probes contained either the native promoter regions, or combinations of WT IR sequences and mutant sequences of polyC.

Proteins were diluted to appropriate concentrations using diluent buffers matching their storage buffer constitution. Each binding reaction contained 2 µl of 5× EMSA binding buffer (750 mM KCl, 50 mM Tris–HCl pH 8.0, 2.5 mM EDTA pH 8.0, 0.5% Triton X-100, 1 mM DTT, 55% glycerol), 250 fmoles of fluorescently labeled probe, 0.1 µl BSA (10 mg/ml), 1 µl poly(d[IC]) (1 mg/ml), 1 µl of diluted protein or buffer control and water to a final volume of 10 µl. Native 0.5× TBE polyacrylamide gels (at either 7% or 5% acrylamide, as required) were pre-run at 150 V and 4°C for 2 h. Binding reactions were titrated at protein concentrations from zero to an appropriate upper limit, and incubated at 20°C for 30 min. Non-specific binding controls used an additional excess of 2.5 µM TRB1108 template DNA amplified with TRB1109 as forward primer, and non-labeled reverse primer. Specific binding controls used additional excess of 2.5 µM unlabeled specific probe DNA. Samples were then separated by native polyacrylamide gel electrophoresis at 200 V and 4°C for 45 min.

Native polyacrylamide gels were then visualized using the Amersham Biosciences Typhoon 9400 on variable mode image in fluorescence mode, emission filter 526 SP. Sensitivity was set to normal. Band intensities were calculated using the grid scan feature and triplicate data processed in Prism (GraphPad Software). Fractional saturation curves were produced with fractional saturation, Y, varying from 0–1.0. Y values are calculated by (Y/(Y + (1 − Y))) and plotted against protein concentration. Data were converted to the Hill plot to analyse the degree of co-operativity in the binding events, characterized by the Hill coefficient (slope of the plot at log(θ) = 0). The Hill plot is constructed by plotting logθ against log[protein], with θ defined as (θ = (Y/(1 − Y))). Dissociation coefficients (*K*_d_) can also be extracted from the Hill plot as *K*_d_ = 10^X − intercept^. Mean and standard error of the mean values are derived from at least three independent experiments.

### Promoter activity assays

Promoter regions were cloned into the promoterless *lacZ* fusion plasmid, pRW50 [32]. Antitoxin genes were cloned into the pQE-80 derivative, pTA100 [5] for tight control of antitoxin expression. Construction is detailed above. Promoter activity assays were performed as described previously [45,46]. Briefly, *E*. *coli* DH5α were co-transformed with the *lacZ* reporter constructs pTRB483 (P*_rv1044_*), pTRB484 (P*_rv2827c_*) or pTRB486 (P*_abiEi_*), and the IPTG-inducible pTA100-antitoxin plasmids pPF656 (Rv1044), pPF658 (Rv2827c) or pTRB481 (AbiEi). Transformants were re-seeded from overnight cultures and grown in 37°C at 200 rpm in LB supplemented with Tc, Sp, and with/without IPTG until mid-log phase, then 80 µl of cells were added to 120 µl master mix (60 mM Na_2_HPO_4_, 40 mM NaH_2_PO_4_, 10 mM KCl, 1 mM MgSO_4_, 36 mM β-mercaptoethanol, 166 µl/ml T7 lysozyme, 1.1 mg/ml ONPG, and 6.7% PopCulture Reagent (Merck Millipore)) in corresponding wells of a 96-well plate. This was then placed in a SPECTROstar Nano absorbance plate reader (BMG LABTECH) set to 30°C with shaking at 500 rpm, wherein OD_600_ and OD_420_ readings were taken every 90 s for 1 hour. Data analysis was performed in the MARS Data Analysis software package (BMG LABTECH). The kinetic OD_420_ readings were converted into the slope of OD_420_ over time (OD_420_/min). These values were multiplied by 5000 and divided by the OD_600_ reading from the first time point to generate Miller Units (mU). Plotted data are the normalized mean and standard deviation obtained from three independent experiments.

## Results

### AbiEi-family antitoxins contain conserved structural features

We had previously hypothesized that there was structural similarity between the biochemically characterized antitoxin AbiEi from *S. agalactiae* [29] and the structurally characterized homolog, Rv2827c [30]. We sought to confirm structural and biochemical similarity between these two proteins, and within the broader COG5340 antitoxins. To begin, we solved a 1.83 Å structure of AbiEi by X-ray crystallography (Figure 1B,C and Table 1). There were four copies of AbiEi in the asymmetric unit, forming minor crystal contacts that are not predicted to be biologically relevant, and each copy contains minor variations in domain orientation, indicating some flexibility. Together with previous size exclusion chromatography data [29], we concluded that AbiEi is a 23 kDa monomer in solution.

AbiEi contains an N-terminal winged helix-turn-helix DNA-binding domain and a C-terminal antitoxin domain, connected by a short linker (Figure 1B). Mutagenesis studies have demonstrated that full-length AbiEi is required for negative autoregulation of the P*_abiE_* promoter, whilst the C-terminal domain alone is sufficient for antitoxicity against the effects of AbiEii [15]. The N-terminal domain contains three α-helices, followed by three beta-strands forming an antiparallel sheet (Figure 1B). The C-terminal domain begins with a single α-helix that is separated from a six-helix bundle by a row of four β-strands, which themselves pair into parallel and antiparallel β-sheets (Figure 1B). One face of AbiEi is positively charged, and the reverse face is negatively charged (Figure 1C). The positive side corresponds with the site of positively charged sidechains distributed throughout the N-terminal and C-terminal domains, which have previously been shown to be vital for DNA-binding and autoregulation through mutagenesis studies [29]. When AbiEi is compared with Rv2827c, both are monomers and it is clear that the two antitoxins share the two-domain structure and charge features (Figure 1B–E).

**Figure 2.**
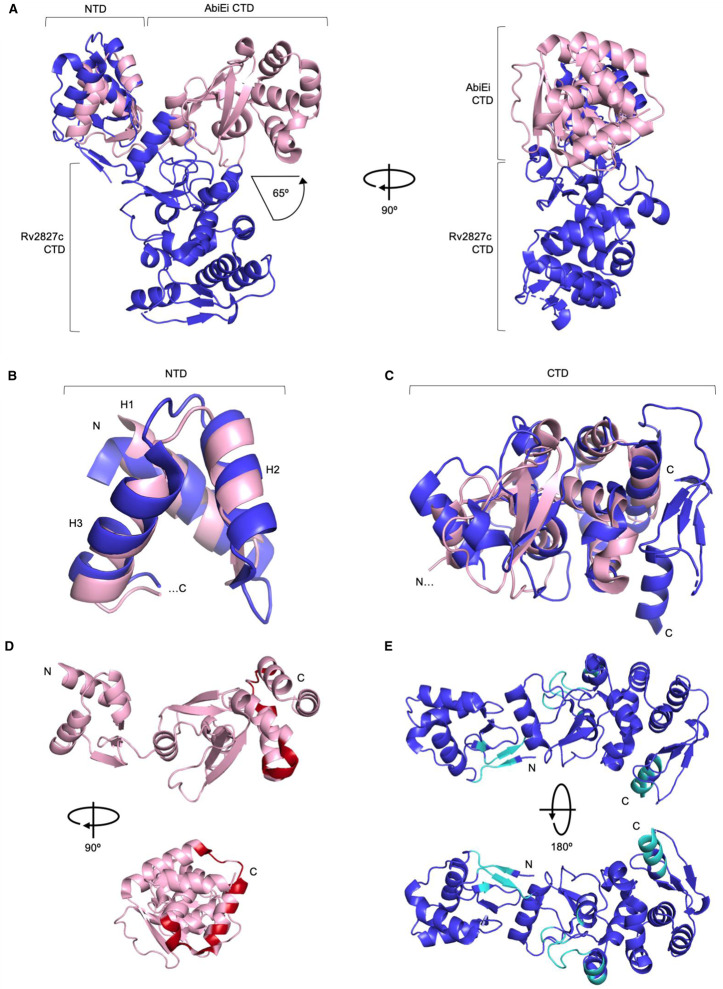
AbiEi and Rv2827c are structurally similar, but have been captured in different positions with differing predicted protein interaction interfaces. (**A**) AbiEi (pink) and Rv2827c (blue) in cartoon representation, aligned via the N-terminal winged helix-turn-helix domains, shown as two orthogonal views. The positions of the C-terminal domains diverge at a 65° angle. (**B**) Close-up structural superposition of the isolated N-terminal helices of AbiEi and Rv2827c, colored as per (**A**). The three helices (H1–3) of the N-terminal winged helix-turn-helix domains align well. (**C**) Close-up structural superposition of the isolated C-terminal domains of AbiEi and Rv2827c, colored as per (**A**). The core secondary structural features of the C-terminal domains approximate to the same positions, but the Rv2827c C-terminal domain has additional features at the C-terminus. (**D**) AbiEi has C-terminal residues predicted to be involved in making protein–protein interactions, which might allow positive co-operativity in AbiEi monomer binding. AbiEi is in pink cartoon representation with identified interacting residues in red, and is shown in orthogonal views. (**E**) Rv2827c does not have an equivalent patch of C-terminal interacting residues. Rv2827c is in blue cartoon representation, with identified interacting residues in cyan, and is shown in 180° rotation. Residues were identified using the cons-PPISP server. Rv2827c PDB code: 1ZEL.

When AbiEi and Rv2827c are aligned via the N-terminal winged helix-turn-helix domain, the respective C-terminal domains differ in position relative to the N-terminal domains by ∼65° (Figure 2A). We propose that these different poses captured in the crystal structures might reflect variable positions of the C-terminal domains potentially allowed by a linker joining the two domains. The stability of the B-factors for the subdomains AbiEi and Rv2827c, alongside lack of significant variation in the domain orientations within the asymmetric unit indicates a preferred state has been captured in the crystal. This however would require further analysis in solution. The extensive nature of the AbiEi charged surface, the requirement for the full AbiEi protein for autoregulation [15], and the presence of a flexible linker altogether indicate the full protein is needed for DNA interactions and DNA bending as per our previously proposed model [29].

When the N-terminal domain helices and C-terminal domains from AbiEi and Rv2827c are separated and structurally superposed, it is possible to see an approximate overlay between corresponding regions, with RMSDs of 3.04 Å for the N-terminal helices and 3.41 Å for the C-terminal domains (Figure 2B,C). The N-terminal domains have conserved positioning of key helices H2 and H3, which are used within winged helix-turn-helix domains for stabilization and DNA recognition, respectively [47] (Figure 2B). The C-terminal domain of AbiEi is the smaller of the two; performing a structure-based sequence alignment of AbiEi and Rv2827c shows that Rv2827c has an extended C-terminal domain 55 amino acids longer than AbiEi (Supplementary Figure S1). Despite this extension, AbiEi and Rv2827c share a conserved common core fold of unknown function (Figure 2C). When AbiEi was compared against the PDB to look for similar structures, using the DALI server [48], Rv2827c was the top hit followed by bacterial antibiotic-modifying adenylyltransferases (PDB codes 5KQJ, 4FO1), and a putative fungal NTase (PDB: 5UVD). These putative biochemical activities for AbiEi match well with the NTase activity of the cognate toxin AbiEii [15]. Overall, despite differing captured poses and discrepancy in size, AbiEi and Rv2827c are markedly similar in domain structure, fold and surface charge and are therefore structural homologs.

It has been shown that the AbiEi C-terminal domain is required for negative autoregulation and likely contributes to positive co-operativity through C-terminal domain interactions [29]. The cons-PPISP server [49] was used to highlight the residues most likely to be critical for protein–protein interactions for both AbiEi and Rv2827c (Figure 2D,E). In the AbiEi monomer, 16 identified residues were clustered at the C-terminus, forming a putative site for interaction (Figure 2D). For Rv2827c, however, the diffuse scattering of 34 identified residues along the structure (Figure 2E) predicts that there may be no obvious interface for protein–protein interactions. This is reinforced by the different positioning of the CTD seen in Rv2827c (Figure 2A). These findings suggest that the interactions of AbiEi C-terminal domains could contribute to positive co-operativity in promoter binding, supporting our previously proposed model, whereas for Rv2827c, such interactions are unlikely to occur and indicate a different mechanism of DNA-binding and autoregulation.

### Rv2827c binds two sets of inverted repeats

**Figure 3.**
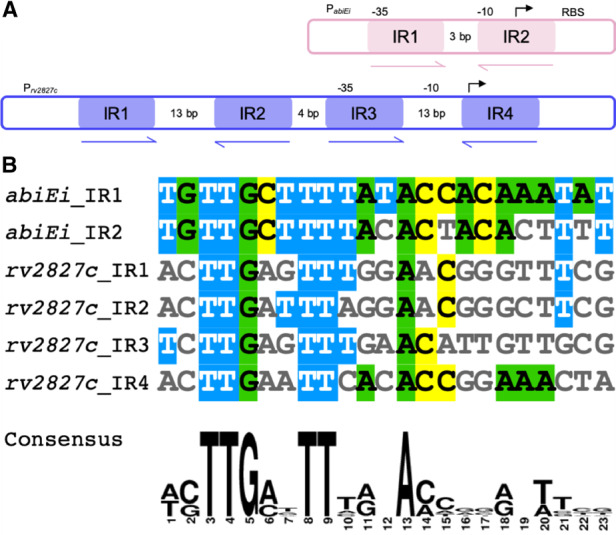
The *rv2827c–rv2826c* promoter has similar features but is more complex than the *abiE* promoter. (**A**) Cartoon of the *abiE* and *rv2827c–rv2826c* promoters (pink and blue, respectively), showing the relative positions of the 23 bp inverted repeats (IRs). Putative transcriptional −35, −10 and start sites, along with ribosome binding sites (RBS), are indicated where possible. (**B**) Alignment of the six, 23 bp, IR sequences shows consensus sequences between the *abiE* and *rv2827c–rv2826c* promoters. The alignment was made using MView and the consensus was made using WebLogo.

AbiEi binds to two 23 bp inverted repeats (IR1 and IR2) within the promoter of P*_abiEi_*, which are separated by 3 bp [29] (Figure 3A). Examination of the upstream region of P*_rv2827c_* revealed two pairs of 23 bp inverted repeats within the region −1 to −131 bp from the *rv2827c* start codon, that also overlap the promoter (Figure 3A). These four repeats (IR1 to IR4) are arranged in tandem with a 4 bp gap between the two pairs of inverted repeats and a 13 bp gap between the repeats within each pair (Figure 3A). As P*_abiEi_* repeats are separated by 3 bp and the repeats within pairs from P*_rv2827c_* are separated by 13 bp, it is possible the additional 10 bp accommodates binding of the larger C-terminal domains of Rv2827c (Supplementary Figure S1). Using the bacterial promoter prediction software CNNPromoter_b [50], the IR3–IR4 repeats were predicted to straddle a binding site for the primary *M. tuberculosis* sigma factor SigA [51]. As Rv2827c binding would sterically hinder sigma factor binding, in turn, this would prevent transcription of the operon by RNA polymerase. When IR1–IR4 sequences from P*_rv2827c_* were aligned with IR1–IR2 sequences from P*_abiEi_*, the sequence similarity indicated possible conservation of binding sequence (Figure 3B). We therefore hypothesized that despite sharing low protein sequence identity (17.7%), Rv2827c might bind these P*_rv2827c_* inverted repeats similarly to AbiEi binding its cognate P*_abiEi_* repeats.

**Figure 4.**
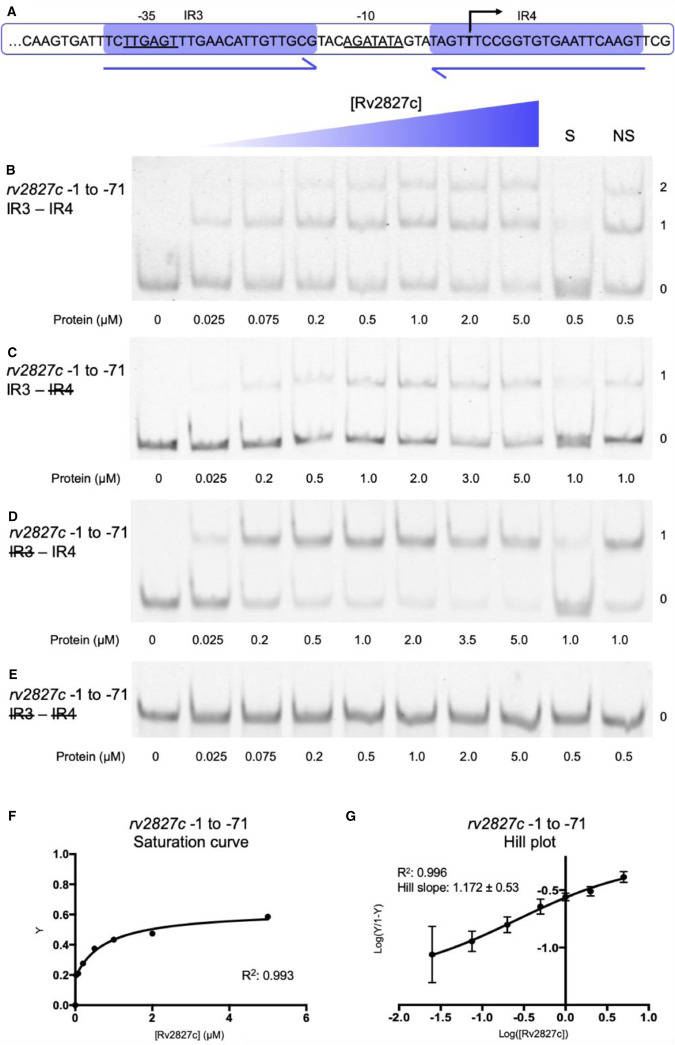
Rv2827c binds non-co-operatively to the IR3–IR4 region of the *rv2827c–rv2826c* promoter. (**A**) Sequence level cartoon of the fluorescently labeled probe containing IR3–IR4, with −35, −10 and transcriptional start indicated. (**B**) Electrophoretic mobility shift assay (EMSA) of titrated Rv2827c with the probe in (**A**). (**C**) EMSA of titrated Rv2827c with the probe in (**A**) altered by replacing IR4 with polyC. (**D**) EMSA of titrated Rv2827c with the probe in (**A**) altered by replacing IR3 with polyC. (**E**) EMSA of titrated Rv2827c with the probe in (A) altered by replacing both IR3 and IR4 with polyC. For (**B**–**E**); protein concentrations are shown on each panel together with the binding events (0, 1 or 2); S — each experiment contained 100-fold excess of the specific unlabeled probe; NS — each experiment contained 100-fold excess of non-specific unlabeled probe; numbering −1 to −71 denotes the promoter region included in the probe, upstream of the translational start site in order to include all of IR4. (**F**) Fractional saturation curve plotted using the EMSA data of (**B**). (**G**) Hill plot using the EMSA data from (**B**). For (**F**) and (**G**), points are plotted from triplicate data and display mean values with standard error of the mean.

The four P*_rv2827c_* 23 bp inverted repeats were first tested as two consecutive pairs, to allow a direct comparison to the arrangement of P*_abiEi_* [29]. Analysis began with IR3–IR4, the pair overlapping the transcriptional start and therefore analogous to IR1–IR2 of P*_abiEi_* (Figure 4A). Using electrophoretic mobility shift assays (EMSAs), Rv2827c was shown to bind both of the IR3–IR4 inverted repeats within the −1 to −71 region (Figure 4B). Sequential removal of the inverted repeats by mutating one, the other or both to polyC tracts reduced Rv2827c-DNA interaction to a single binding event (Figure 4C,D) or ablated binding completely (Figure 4E). Analysis of IR3–IR4 binding (Figure 4B,F) showed weak saturation of binding. The calculated Hill coefficient indicates that IR3–IR4 binding by Rv2827c is not co-operative (Figure 4G).

**Figure 5.**
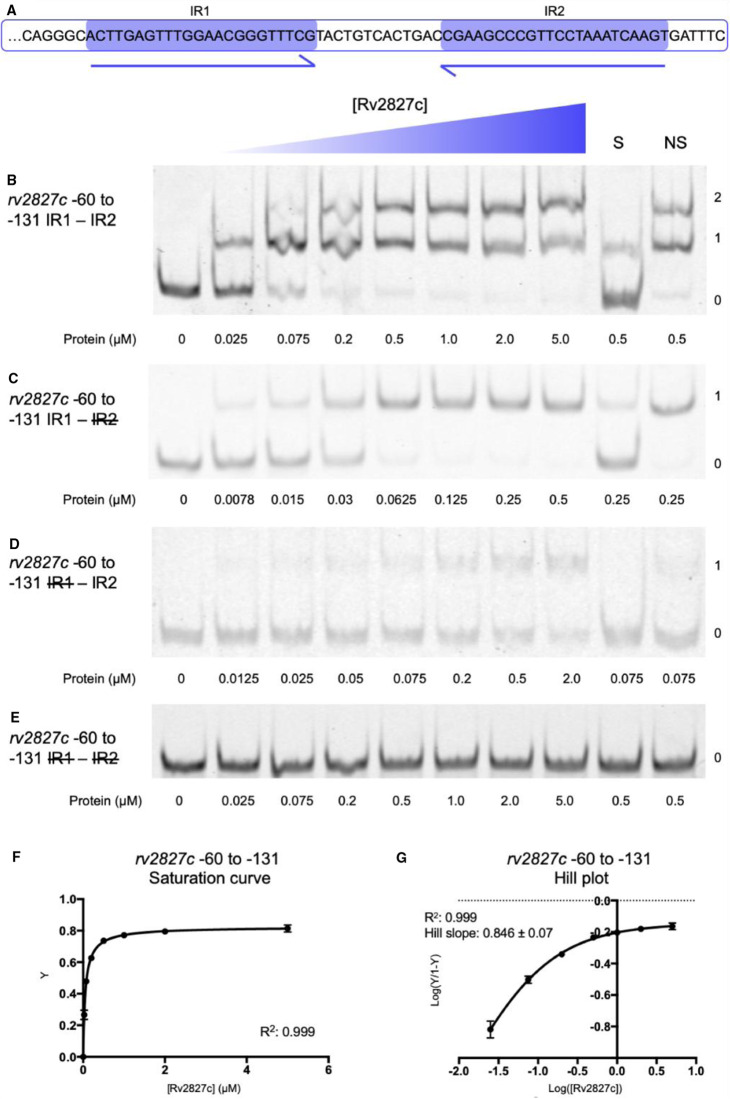
Rv2827c binds with weak negative co-operativity to the IR1–IR2 region of the *rv2827c–rv2826c* promoter. (**A**) Sequence level cartoon of the fluorescently labeled probe containing IR1–IR2. (**B**) Electrophoretic mobility shift assay (EMSA) of titrated Rv2827c with the probe in (**A**). (**C**) EMSA of titrated Rv2827c with the probe in (**A**) altered by replacing IR2 with polyC. (**D**) EMSA of titrated Rv2827c with the probe in (**A**) altered by replacing IR1 with polyC. (**E**) EMSA of titrated Rv2827c with the probe in (**A**) altered by replacing both IR1 and IR2 with polyC. For (**B**–**E**); protein concentrations are shown on each panel together with the binding events (0, 1 or 2); S — each experiment contained 100-fold excess of the specific unlabeled probe; NS — each experiment contained 100-fold excess of non-specific unlabeled probe; numbering −60 to −131 denotes the promoter region included in the probe. (**F**) Fractional saturation curve plotted using the EMSA data of (**B**). (**G**) Hill plot using the EMSA data from (**B**). For (**F**) and (**G**), points are plotted from triplicate data and display mean values with standard error of the mean.

Similar results were obtained when testing the IR1–IR2 repeats within the −61 to −131 region of P*_rv2827c_* (Figure 5A–G). In this case, there was greater saturation of binding to IR1–IR2 (Figure 5F) than to IR3–IR4 (Figure 4F). The Hill coefficient surprisingly indicated weakly negative co-operativity in Rv2827c binding to IR1 and IR2 (Figure 5G), in comparison with the non-co-operative binding observed with IR3 and IR4 (Figure 4G). To allow direct comparison between model systems, we also performed the same assays with purified AbiEi and probes for P*_abiEi_* (Supplementary Figure S2). This corroborated previous data [29] and under our experimental conditions, AbiEi bound more tightly to its cognate inverted repeats (Supplementary Figure S2F), than either Rv2827c binding to IR3–IR4 (Figure 4F) or IR1–IR2 (Figure 5F), and also demonstrated clear positive co-operativity (Supplementary Figure S2G).

Due to similarity of structure, functionality and cognate DNA inverted repeat sequences, we hypothesized that AbiEi and Rv2827c might bind their respective non-cognate promoter regions. However, AbiEi did not bind either pair of inverted repeats from P*_rv2827c_* (Supplementary Figure S3A,B). Similarly, Rv2827c did not bind IR1–IR2 of P*_abiEi_* (Supplementary Figure S3C).

### Rv2827c binds with negative co-operativity

**Figure 6.**
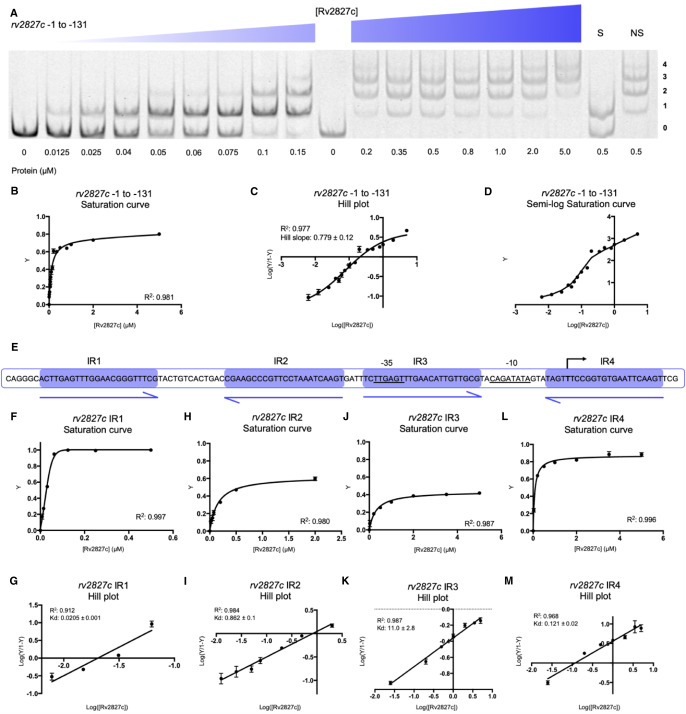
Rv2827c binds with negative co-operativity to the full *rv2827c–rv2826c* promoter. (**A**) EMSA of titrated Rv2827c with a probe covering from −1 to −131 of the r*v2827c–rv2826c* promoter, covering IR1 to IR4. The titration was performed across two EMSA gels, with an additional zero protein lane included in the second gel for normalization. Protein concentrations are shown below each gel together with the binding events (0, 1, 2, 3 or 4); S — each experiment contained 100-fold excess of the specific unlabeled probe; NS — each experiment contained 100-fold excess of non-specific unlabeled probe. (**B**) Fractional saturation curve plotted using the EMSA data of (**A**). (**C**) Hill plot using the EMSA data from (**A**). (**D**) Semi-log saturation curve plotted using the EMSA data of (**A**), showing distinct breaks in the binding curve, in accordance with the multiple binding sites contained in the probe. (**E**) Sequence level cartoon of the fluorescently labeled probe containing r*v2827c–rv2826c* −1 to −131. (**F**–**M**) Saturation curves (**F**,**H**,**J**,**L**) and Hill plots (**G**,**I**,**K**,**M**) for each IR calculated using individual IR data gathered using mutant probes (Figures 4C, D and 5C,D). For (**B**–**D**) and (**F**–**M**), points are plotted from triplicate data and display mean values with standard error of the mean.

Having investigated the two sets of P*_rv2827c_* inverted repeats independently, a full-length probe covering the P*_rv2827c_* region −1 to −131 was generated to examine the interaction of Rv2827c protein with all four inverted repeats. Using EMSAs, four distinct protein-bound DNA species were observed, indicating that all four inverted repeats can be bound simultaneously by Rv2827c (Figure 6A). The four binding sites did not fully saturate (Figure 6B), and the Hill coefficient confirmed negatively co-operative binding of Rv2827c across these four inverted repeats (Figure 6C). Displaying the saturation curve data on a semi-log scale highlights breaks and multiple distinct gradients in the binding curve, eluding to multiple individual binding events (Figure 6D). Negatively co-operative binding by Rv2827c to P*_rv2827c_* contrasts with the positive co-operativity observed for AbiEi binding to P*_abiEi_* [29].

Our earlier data using mutant probes provided insight into how Rv2827c binds to individual repeats (Figures 4C,D and 5C,D). These were used to calculate the binding affinity of Rv2827c for each individual IR sequence, with IR1 most tightly bound (*K*_d_ of 0.0205 µM), closely followed by IR4 (*K*_d_ of 0.121 µM), then IR2 (*K*_d_ of 0.862 µM), and finally IR3 (*K*_d_ of 11.0 µM) (Figure 6E–M). This descending affinity series creates a wide range of concentrations across which negative autoregulation by Rv2827c can occur. These data demonstrate the same core principles of promoter binding are used by both AbiEi and Rv2827c, but that these have been employed evolutionarily for differing modes of regulation.

### Rv1044 is a DNA-binding protein, but fails to recognize the cognate promoter

Whilst it had not been possible to identify inverted repeats within the *rv1044*–*rv1045* locus, two, slightly overlapping, 70 bp probes were generated to cover the 131 bp region upstream of the *rv1044* translational start site, and used to test Rv1044 binding (Supplementary Figure S4A,B). No binding event was observed with either probe (Supplementary Figure S4A,B). Nevertheless, we wanted to test whether Rv1044 was competent for DNA-binding and so cross-reacted Rv1044 with the two probes covering IR3–IR4 and IR1–IR2 of P*_rv2827c_*, and the probe containing IR1–IR2 of P*_abiEi_* (Supplementary Figure S4C–E). No binding was observed for either of the P*_rv2827c_* probes (Supplementary Figure S4C,D), but curiously, Rv1044 bound the inverted repeats of P*_abiEi_* (Supplementary Figure S4E). Rv1044 bound more weakly than AbiEi to P*_abiEi_* IR1–IR2 (Supplementary Figure S4F), and showed no co-operativity (Supplementary Figure S4G). This demonstrates that Rv1044 can bind DNA in a sequence-specific manner, and so we looked for potential targets in the *M. tuberculosis* H37Rv genome. The *abiE* IR sequences align with numerous positions in the *M. tuberculosis* genome but not upstream of any of the DUF1814 TA systems. This may indicate a potential role for Rv1044 in regulating genes outside of the *rv1044–rv1045* operon, as has been shown in other TA systems whereby antitoxins influence gene expression in biofilm formation pathways [52–54]. A further study will be needed to fully explore any potential regulatory role of Rv1044.

### Rv2827c negatively autoregulates Rv2827c–Rv2826c expression

**Figure 7.**
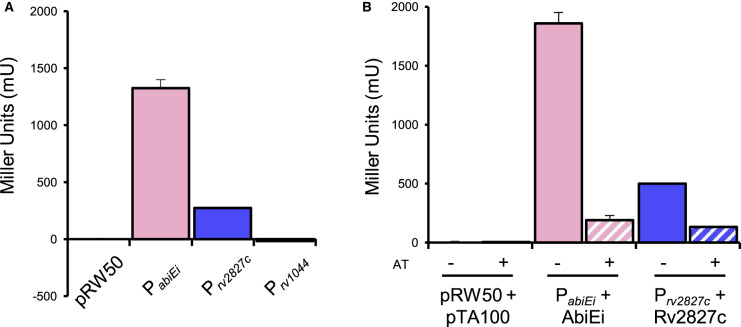
Rv2827c–Rv2826c is a negatively autoregulating system in *E. coli*. (**A**) Promoter activity from upstream promoter regions of *abiE* (99 bp), and *rv2827c–rv2826c* and *rv1044–rv1045* (500 bp for both) detected using *lacZ* transcriptional fusions. Both the *abiE* and *rv2827c–rv2826c* constructs are active, but the *rv1044–rv1045* construct is not. Plotted data are normalized to the vector-only control. (**B**) Autoregulation of promoter activity by antitoxins. LacZ activity was measured from the *abiE* and *rv2827c–rv2826c* constructs with or without induction of the cognate antitoxin (AT, ±IPTG). Both AbiE and Rv2827c negatively autoregulate expression. Plotted data are normalized to the uninduced vector-only control. All data (**A**–**B**) are plotted as the means of triplicate data, and error bars show standard deviations from the mean.

Having shown a structural similarity between the two antitoxins, we next sought to test whether the COG5340 proteins from *M. tuberculosis* could function as autoregulators, like characterized AbiEi [29]. AbiEi negatively autoregulates expression from the P*_abiEi_* promoter [29]. To examine whether Rv2827c and also the second *M. tuberculosis* COG5340 protein, Rv1044, also perform negative autoregulation, we first cloned the 500 bp region upstream of each respective translational start site into a promoterless *lacZ*-reporter plasmid [32]. For comparison, the equivalent P*_abiEi_*–reporter, containing the previously identified promoter region identified in the upstream 99 bp [15,29] was also tested. Both P*_abiEi_* and P*_rv2827c_* reporters yielded expression of LacZ, but P*_rv1044_* did not (Figure 7A). The two active reporter constructs P*_abiEi_* and P*_rv2827c_*, were then paired with inducible plasmids for expression of the cognate antitoxins, and LacZ levels were determined with and without antitoxin induction (Figure 7B). When compared with the uninduced controls, both antitoxins negatively autoregulated expression from their cognate promoters (Figure 7B) demonstrating that Rv2827c and AbiEi share not only a common structure, but also a common negative autoregulatory function.

## Discussion

In this study we present the crystal structure of *S. agalactiae* AbiEi, which was the first type IV TA system antitoxin shown to be capable of transcriptional autoregulation through promoter binding [29]. Further to this, we have demonstrated the autoregulatory capacity of the related Rv2827c antitoxin, a protein of known structure [30]. Whilst AbiEi is a structural homolog of the Rv2827c antitoxin, and both share similar promoter architectures, they have distinct differences in their size and captured domain orientations. We also show that negative autoregulation of the P*_rv2827c_* promoter operates via negatively co-operative interactions.

**Figure 8.**
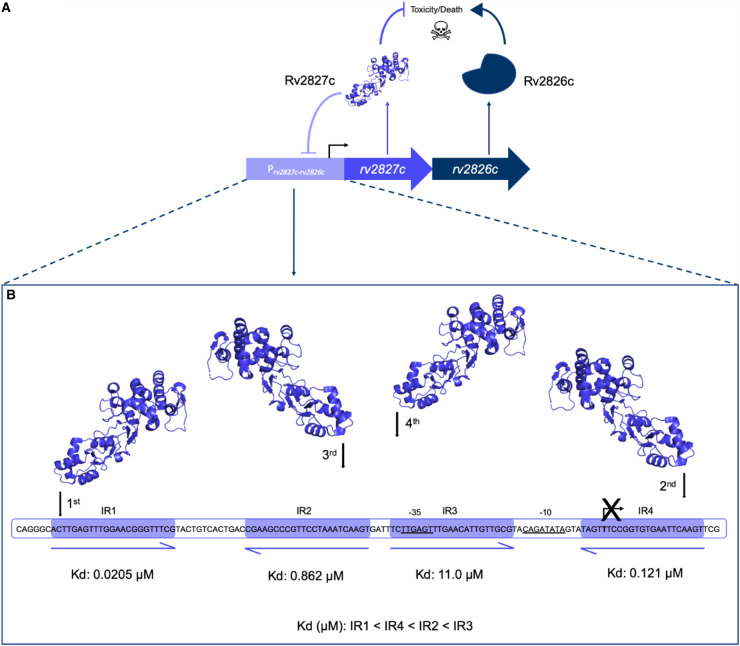
Proposed model for negative autoregulation caused by Rv2827c binding to the four *rv2827c–rv2826c* promoter inverted repeats. (**A**) Schematic representation of the putative rv2827c–rv2826c type IV toxin-antitoxin system. Model shows both *rv2827c* and *rv2826c* being translated into the antagonistic antitoxin and toxin protein pair, respectively. The antitoxin, Rv2827c, has a second function and binds to the *rv2827c–rv2826c* promoter, negatively autoregulating the operon. (**B**) An order of binding is created by the distinct affinity values for the inverted repeats represented in the sequence level cartoon, calculated from individual IR data gathered using mutant probes (Figures 4C,D and 5C, D). Rv2827c binds negatively co-operatively, initially to IR1 (0.0205 µM) followed by IR4 (0.121 µM), IR2 (0.862 µM) and finally IR3 (11.0 µM).

Despite the low shared sequence similarity seen for the COG5340 antitoxins investigated (AbiEi and Rv2827c — 17.7%; AbiEi and Rv1044 — 21.2%; Rv2827c and Rv1044 — 24.5%), we have demonstrated structural conservation across species. As sequences diverge, structure is conserved (Figures 1, 2), which maintains the shared functionality of these antitoxins, for instance, DNA-binding (Figures 4–7 and Supplementary Figures S2–S4). Interestingly, sequence variation of the NTD, alongside differing promoter architectures, has resulted in at least three variations of antitoxicity. AbiEi and Rv2827c both autoregulate their own operons, albeit with contrasting types of co-operativity. Rv1044, however, may regulate genes elsewhere in the *M. tuberculosis* genome, given the lack of affinity to the *rv1044* upstream region tested (Supplementary Figure S4A,B) and absence of identifiable inverted repeats, but apparent DNA-binding capabilities (Supplementary Figure S4E–G). Further analysis will be required to identify a functional promoter for the *rv1044–rv1045* operon and confirm any potential regulatory function of Rv1044. The antitoxic CTDs have a common core fold that are predicted to have NTase activity based on structure-based functional searches [30]. Therefore, the antitoxic mechanism is likely conserved, despite low sequence similarity within these domains (Supplementary Figure S1). As protein sequences will be tuned to the needs of the organism, we have shown a correspondingly differential pattern of residues for protein–protein interactions (Figure 2D,E) which, alongside the different CTD positions captured (Figure 2A), may contribute to individual autoregulation requirements. Our previous model predicted AbiEi C-terminal domain interactions promote positive co-operative binding and result in DNA-bending [29], however this does not appear to apply to Rv2827c. Our proposed model (Figure 8) implies a possible lack of protein–protein interactions supported by predicted interaction interfaces (Figure 2D,E), while not ruling out the potential for steric restriction. Rather, promoter inverted repeat sequence ‘tuning’ (Figure 3) contributes to the negatively co-operative interaction via descending affinities.

Promoters of *M. tuberculosis* are known to be more complex than those of *E. coli*; they can stretch to 2000 bp from the start site and lack canonical elements such as the conserved −35 sequence [55–57]. Transcriptional regulation is complicated further when considering the vast number of sigma factors [58] and environmentally responsive transcription factors [59] present in *M. tuberculosis*, allowing for greater promoter sequence variation. The −10 sequence for *rv2827c–rv2826c* is a predicted recognition site for principle *M. tuberculosis* sigma factor SigA, which is usually maintained at a constant level for cellular ‘housekeeping' [51]. SigA also has a role in host-pathogen interactions, controlling growth rates during macrophage infection [60] and regulating virulence genes through both constitutive and up-regulated expression [61–63]. Deletions of *rv2827c* cause a growth defect [25,26], suggesting SigA drives expression and that there is potential for output to be tuned by SigA and Rv2827c levels according to environmental cues. Previous reports on the type IV antitoxin CbeA demonstrate a positive effect on cytoskeletal bundling alongside antitoxicity and the ability to counteract chemical inhibitors of cytoskeletal polymerisation [13]. One study has shown Rv2827c up-regulation in response to isoniazid and rifampicin treatment, albeit as part of more general TA system up-regulation [64].

The IR conservation between P*_abiEi_* and P*_rv2827c_* (Figure 3) suggested that autoregulation may also occur in a biochemically comparable manner between the two. However, Rv2827c bound the pairs of inverted repeats with either no co-operativity (Figure 4), or weakly negative co-operativity (Figure 5). There was clear negative co-operativity when all four inverted repeats were tested (Figure 6). Analyzing each inverted repeat independently by mutational studies identified significant differences between the Rv2827c-IR dissociation constants (Figure 6E–M). These data have allowed us to propose a model for the regulation of *rv2827c–rv2826c* (Figure 8). As *rv2827c* is needed for normal growth, this suggests that *rv2826c* encodes a toxin capable of causing growth defects [25,65], which is antagonized by Rv2827c (Figure 8A). Expression of *rv2827c–rv2826c* is negatively autoregulated by Rv2827c, and this is made possible by sequential binding of Rv2827c to the four IR sequences, in order as determined by binding affinity (Figure 8B). Given the high concentration of Rv2827c required to saturate the lower affinity site IR3 (Figures 6J,K and 8B), mimicking the mutational analysis performed here in promoter activity studies would provide useful insight into the function of each IR sequence. These binding events have apparent negative co-operativity, likely due to the variations in IR sequences creating a series of binding steps with ever-decreasing affinity. To better understand these negatively co-operative interactions further experiments are required, exploring the role of the Rv2827c CTD and larger inverted repeat spacers, akin to previous work on AbiEi [29].

Negative co-operativity was an unexpected result given the structural similarities between the N-terminal domains of AbiEi and Rv2827c (Figure 2B), and the similarities of their respective promoter architectures (Figure 3). Examples of negative and positive co-operativity have been found in equal abundance across all organisms [66,67]. Positive co-operativity leads to rapid saturation at a defined, short range of concentrations as seen for *abiE* [29]. In contrast, negative co-operativity of Rv2827c binding would be expected to generate a relatively delayed response, working across a greater range of Rv2827c concentrations [67–69]. This variability in tuning according to concentration could in turn relate to the relative potency of the toxins and dosage required to have an effect in their cognate hosts. This variation is evident when comparing saturation curves of AbiEi and Rv2827c to their cognate full-length promoters (Figure 6B and Supplementary Figure S2F). Compared with positive co-operativity, there is relatively little information on the presence of negatively co-operative TA-promoter interactions. However, clear evidence supports weaker binding of un-complexed type II antitoxins [52,70] when compared with the conditionally co-operative binding of TA complexes [28,52,71,72]. It is noteworthy that unlike many type II antitoxins, AbiEi and Rv2827c are fully folded and stable, and also no conditionally co-operative response was seen for AbiE, and so the conditional model proposed for many type II systems likely does not apply [15].

This study has shown that the similar structures and promoter architectures between AbiEi, Rv2827c (and indeed Rv1044) have been co-opted to form different regulatory modules. A greater understanding of how these nuances of regulation are applied in the cognate hosts may provide greater insight into the control of bacterial growth. Understanding these systems and how they regulate bacterial behavior is thereby an important step in developing a means to control TA systems towards utilizing them for their potential therapeutic value.

## Data Availability

The crystal structure of AbiEi has been deposited in the Protein Data Bank under accession number 6Y8Q.

## Abbreviations

CTDs: C-terminal domains
EMSAs: electrophoretic mobility shift assays
IPTG: isopropyl-β-D-thiogalactopyranoside
LIC: Ligation Independent Cloning
NTase: nucleotidyltransferase
