# Supplementary material for: Antitoxin autoregulation of M. tuberculosis toxin-antitoxin expression through negative cooperativity arising from multiple inverted repeat sequences
Source: Biochem J. 2020 Jun 26;477(12):2401–19. doi: 10.1042/BCJ20200368 (PMC7319586; doi:10.1042/BCJ20200368)
Supplement: Supplementary Figures S1-S4 and Tables S1-S2 [file BCJ-477-2401-s1.pdf]

Supplementary data

Supplementary Figures

Fig. S1. AbiEi and Rv2827c have similar folds. Structure-based sequence alignment of AbiEi and Rv2827c, drawn by hand using output from PROMALS3D. Blue arrows represent β-sheets and pink ovals represent α-helices; numbers indicate amino acid positions.

Fig. S2. AbiEi binds with positive co-operativity to the IR1-IR2 region of the *abiE* promoter. (A) Sequence level cartoon of the fluorescently labelled probe containing IR1-IR2, with -35, -10, transcriptional start and ribosome binding site (RBS) indicated. (B) Electrophoretic mobility shift assay (EMSA) of titrated AbiEi with the probe in (A). (C) EMSA of titrated AbiEi with the probe in (A) altered by replacing IR2 with polyC. (D) EMSA of titrated AbiEi with the probe in (A) altered by replacing IR1 with polyC. (E) EMSA of titrated AbiEi with the probe in (A) altered by replacing both IR1 and IR2 with polyC. For (B-E); protein concentrations are shown on each panel together with the binding events (0, 1 or 2); S – each experiment contained 100-fold excess of the specific unlabelled probe; NS – each experiment contained 100-fold excess of non-specific unlabelled probe; numbering -1 to -71 denotes the promoter region included in the probe, upstream of the translational start site in order to include all of IR2. (F) Fractional saturation curve plotted using the EMSA data of (B). (G) Hill plot using the EMSA data from (B). For (F) and (G), points are plotted from triplicate data and display mean values with standard error of the mean.

Fig. S3. AbiEi and Rv2827c do not bind non-cognate promoters. (A) Electrophoretic mobility shift assay (EMSA) of titrated AbiEi with *rv2827c-rv2826c* promoter -1 to -71. (B) EMSA of titrated AbiEi with *rv2827c-rv2826c* promoter -61 to -131. (C) EMSA of titrated Rv2827c with *abiE* promoter -1 to -71. For (A-C); protein concentrations are shown below (C) together with the binding events (0, 1 or 2); S – each experiment contained 100-fold excess of the specific unlabelled probe; NS – each experiment contained 100-fold excess of non-specific unlabelled probe; numbering denotes the promoter region included in the probe, upstream of the translational start site in order to include all of the respective IR sequences.

Fig. S4. Rv1044 does not bind the cognate promoter but is capable of DNA-binding. (A) EMSA of titrated Rv1044 with *rv1044-rv1045* promoter -1 to -71. (B) EMSA of titrated Rv1044 with *rv1044-rv1045* promoter -61 to -131. (C) EMSA of titrated Rv1044 with *rv2827c-rv2826c* promoter -1 to -71. (D) EMSA of titrated Rv1044 with *rv2827c-rv2826c* promoter -61 to -131. (E) EMSA of titrated Rv1044 with *abiE* promoter -1 to -71. For (A-E); protein concentrations are shown on each panel together with the binding events (0, 1 or 2); S – each experiment contained 100-fold excess of the specific unlabelled probe; NS – each experiment contained 100-fold excess of non-specific unlabelled probe; numbering denotes the promoter region included in the probe, upstream of the translational start site in order to include all of the respective IR sequences. (F) Fractional saturation

curve plotted using the EMSA data of (E). (G) Hill plot using the EMSA data from (E). For (F) and (G), points are plotted from triplicate data and display mean values with standard error of the mean.

SUPPLEMENTARY TABLES

Table S1. Oligonucleotides used in this study

Primer	Sequence[a]	Notes (Organism/Gene)
**pRW50 cloning**		
TRB1072	TTTGAATTCGATTTTGTTATCACAATAAATTGAGG	FWD EcoRI, 99 bp upstream of *abiEi*, *S. agalactiae*
TRB1047	TTTAAGCTTTACGGCCCCCACTTGTTGC	REV HindIII, 99 bp upstream of *abiEi*, *S. agalactiae*
TRB1042	TTTGAATTCGCCCAAGCATCGGCTGGC	FWD EcoRI, 500 bp upstream of *rv2827c*, *M. tuberculosis*
TRB1043	TTTAAGCTTCCGAACTTGAATTCACACCGG	REV HindIII, 500 bp upstream of *rv2827c*, *M. tuberculosis*
TRB1040	TTTGAATTCGGGTCCCAACCGAGCGGC	FWD EcoRI, 500 bp upstream of *rv1044*, *M. tuberculosis*
TRB1041	TTTAAGCTTATTAGGTGATGGAGGCCAAGGCC	REV HindIII, 500 bp upstream of *rv1044*, *M. tuberculosis*
**pSAT1-LIC cloning**		
TRB873	TTAATGCAGCTGATTAATACG	FWD pSAT LIC sequencing
TRB875	TACTCAAGCTTATGCATGC	REV pSAT LIC sequencing
TRB1048	CAACAGCAGACGGGAGGTTCAAAAAAAGAGATTCTACTCGATTTTATAG	FWD *abiEi* LIC, *S. agalactiae*
TRB1049	GCGAGAACCAAGGAAAGGTTATTATATTAGAACCTCCAGAGTTTGTTTAAC	REV *abiEi* LIC, *S. agalactiae*
TRB1022	CAACAGCAGACGGGAGGTGTGAGCCCAGCCGGCGCC	FWD *rv2827c* LIC, *M. tuberculosis*

TRB1023	GCGAGAACCAAGGAAAGGTTATTACGCCTTGC CGATCACGCGCAGC	REV *rv2827c* LIC, *M. tuberculosis*
TRB1018	CAACAGCAGACGGGAGGTTGTGCAAAACCGT ATCTAATTGATACGATTGCGC	FWD *rv1044* LIC, *M. tuberculosis*
TRB1019	GCGAGAACCAAGGAAAGGTTATTACGCCGATG CTCGCTTCGG	REV *rv1044* LIC, *M. tuberculosis*

pTA100 cloning

TRB1052	TTTGAATTCAGGAGGACAGGGATGTCAAAAAA AGAGATTCTACTC	FWD EcoRI, *abiEi*, *S. agalactiae*
TRB1053	TTTAAGCTTGGTTATTATATTAGAACCTCCAGA GTTTG	REV HindIII, *abiEi*, *S. agalactiae*
PF1334	TTTCATATGCAATTGAGGAGGACAGGGATGGT GAGCCCAGCCG	FWD NdeI/MfeI, *rv2827c, M. tuberculosis*
PF1335	TTTACTAGTCCCGGGGTCACGCCTTGCCGATC	REV SpeI/XmaI, *rv2827c, M. tuberculosis*
PF1330	TTTCATATGCAATTGAGGAGGACAGGGATGTG TGCAAAACCGTATCTAA	FWD NdeI/MfeI, *rv1044, M. tuberculosis*
PF1331	TTTACTAGTCCCGGGCTTGGTCACGCCGATG	REV SpeI/XmaI, *rv1044, M. tuberculosis*

EMSA probe primers and templates

TRB1067	TGCGCACTGACAAAAGCTT	REV EMSA untagged
TRB1068	/56-FAM/TGCGCACTGACAAAAGCTT	REV EMSA 56-FAM (fluorescein) tagged
TRB1061	AAAAGAAAATGTTGCTTTTATACCACAAATATT GTAAAATTGTAGTGTAAAAGCAACAAGTGGGG GGCCGTAAGCTTTTGTCAGTGCGCA	*S. agalactiae / abiEi -1 to -71* WT (**Fig. S2B**, **Fig. S3C**, **Fig. S4E**)
TRB1065	AAAAGAAAATGTTGCTTTTATACCACA	FWD for TRB1061, TRB1063, *S. agalactiae, abiEi*
TRB1062	AAAAGAAAACCCCCCCCCCTACCACAAATATT GTAAAATTGTAGTGTAAAAGCAACAAGTGGGG GGCCGTAAGCTTTTGTCAGTGCGCA	*S. agalactiae / abiEi -1 to -71* Mutant; inverted repeat 1 poly-C track substitution (**Fig. S2D**)
TRB1066	AAAAGAAAACCCCCCCC	FWD for TRB1062, TRB1064, *S. agalactiae, abiEi*

TRB1063	AAAAGAAAATGTTGCTTTTATACCACAAATATTGTAAAATTGTAGTGCCCCCCCCCCCAGTGGGGGGCCGTAAGCTTTTGTCAGTGCGCA	*S. agalactiae / abiEi -1 to -71* Mutant; inverted repeat 2 poly-C track substitution (**Fig. S2C**)
TRB1064	AAAAGAAAACCCCCCCCCCCTACCACAAATATTGTAAAATTGTAGTGCCCCCCCCCCCAGTGGGGGGCCGTAAGCTTTTGTCAGTGCGCA	*S. agalactiae / abiEi -1 to -71* Mutant; inverted repeat 1 & 2 poly-C track substitution (**Fig. S2E**)
TRB1086	AACTAGGCGCGCCTAGCCTGGACGAGTCCCCGGGCCGACATTCGCCCGAGGCCTTGGCCTCCATCACCTAAAAGCTTTTGTCAGTGCGCA	*M. tuberculosis* H37Rv */ rv1044, -1 to -71* WT (**Fig. S4A**)
TRB1087	AACTAGGCGCGCCTAG	FWD for TRB1086, *M. tuberculosis, rv1044*
TRB1102	GTATCTGCGACAAGGGCAGCGTCGATGCCTCGACATGCAGAGTCGGTGTTCGCTTCACGCGAACTAGGCGCAAGCTTTTGTCAGTGCGCA	*M. tuberculosis* H37Rv */ rv1044, -61 to -131* WT (**Fig. S4B**)
TRB1103	GTATCTGCGACAAGGGCAG	FWD for TRB1102, *M. tuberculosis, rv1044*
TRB1104	CAAGTGATTTCTTGAGTTTGAACATTGTTGCGTACAGATATAGTATAGTTTCCGGTGTGAATTCAAGTTCGAAGCTTTTGTCAGTGCGCA	*M. tuberculosis* H37Rv */ rv2827c, -1 to -71* WT (**Fig. 4B**, **Fig. S3A, Fig. S4C**)
TRB1105	CAAGTGATTTCTTGAGTTTGAACATTG	FWD for TRB1104, TRB1271, *M. tuberculosis, rv2827c*
TRB1106	CAGGGCACTTGAGTTTGGAACGGGTTTCGTACTGTCACTGACCGAAGCCCGTTCCTAAATCAAGTGATTTCAAGCTTTTGTCAGTGCGCA	*M. tuberculosis* H37Rv */ rv2827c, -61 to -131* WT (**Fig. 5B, Fig. S3B, Fig. S4D**)
TRB1107	CAGGGCACTTGAGTTTGGAAC	FWD for TRB1106, TRB1277, full-length *-1 to -131* (**Fig. 6A**), *M. tuberculosis, rv2827c*
TRB1108	TGGCATTCAATCGATGGCTTCCTAGTTTTAGATGATTAGGGCTTGTCCCAAATGGATTGAGAGGTTGACAAAGCTTTTGTCAGTGCGCA	Plasmid pEFER, 12851-12920 bp NS probe (**Fig. 4 – 6, Fig. S2 – S4**)
TRB1109	TGGCATTCAATCGATGGCTT	FWD plasmid pEFER NS probe
TRB1271	CAAGTGATTCCCCCCCCCCCCCCCCCCCCCCCTACAGATATAGTATAGTTTCCGGTGTGAATTCAAGTTCGAAGCTTTTGTCAGTGCGCA	*M. tuberculosis* H37Rv */ rv2827c, -1 to -71* Mutant; inverted repeat 3 poly-C track substitution (**Fig. 4D**)
TRB1272	CAAGTGATTCCCCCCCCC	FWD for TRB1271, TRB1274, *M. tuberculosis, rv2827c*
TRB1273	CAAGTGATTTCTTGAGTTTGAACATTGTTGCGTACAGATATAGTACCCCCCCCCCCCCCCCCCCCC	*M. tuberculosis* H37Rv */ rv2827c, -1 to -71* Mutant; inverted repeat 4

	CCTCGAAGCTTTTGTCAGTGCGCA	poly-C track substitution (**Fig. 4C**)
TRB1274	CAAGTGATTCCCCCCCCCCCCCCCCCCCCCCCTA CAGATATAGTACCCCCCCCCCCCCCCCCCCCCC CTCGAAGCTTTTGTCAGTGCGCA	*M. tuberculosis* H37Rv */ rv2827c, -1 to -71* Mutant; inverted repeat 3 & 4 poly-C track substitution (**Fig. 4E**)
TRB1275	CAGGGCCCCCCCCCCCCCCCCCCCCCCCCTACT GTCACTGACCGAAGCCCGTTCCTAAATCAAGT GATTTAAGCTTTTGTCAGTGCGCA	*M. tuberculosis* H37Rv */ rv2827c, -61 to -131* Mutant; inverted repeat 1 poly-C track substitution (**Fig. 5D**)
TRB1276	CAGGGCCCCCCCCCC	FWD for TRB1275, TRB1278, *M. tuberculosis, rv2827c*
TRB1277	CAGGGCACTTGAGTTTGGAACGGGTTTCGTAC TGTCACTGACCCCCCCCCCCCCCCCCCCCCCCCC GATTTAAGCTTTTGTCAGTGCGCA	*M. tuberculosis* H37Rv */ rv2827c, -61 to -131* Mutant; inverted repeat 2 poly-C track substitution (**Fig. 5C**)
TRB1278	CAGGGCCCCCCCCCCCCCCCCCCCCCCCCTACT GTCACTGACCCCCCCCCCCCCCCCCCCCCCCCG ATTTAAGCTTTTGTCAGTGCGCA	*M. tuberculosis* H37Rv */ rv2827c, -61 to -131* Mutant; inverted repeat 1 & 2 poly-C track substitution (**Fig. 5E**)

[a]EMSA probe sequences are fused with a constant region from the *lacZ* gene of pRW50, highlighted in grey. The reverse primers (TRB1067 and TRB1069) anneal to this sequence for amplification.

Table S2. Plasmids used in this study

Plasmid	Construct	Cloning technique	Primer set/Restriction enzymes used	Reference
pPF656	pTA100-*rv1044*	Restriction Cloning	PF1330/PF1331	This work
pPF658	pTA100-*rv2827c*	Restriction Cloning	PF1334/PF1335	This work
pRLD30	pTRB30-His_6-*abiEi*	-	-	[15]
pRW50	Tc^R	-	-	[32]
pSAT1-LIC	Ap^R	-	-	This work
pTA100	Sm^R	-	-	[5]
pTRB481	pTA100-*abiEi*	Restriction Cloning	TRB1052/TRB1053	This work
pTRB483	pRW50-500 bp upstream *rv1044*	Restriction Cloning	TRB1040/TRB1041	This work
pTRB484	pRW50-500 bp upstream *rv2827c*	Restriction Cloning	TRB1042/TRB1043	This work
pTRB486	pRW50-99 bp upstream *abiEi*	Restriction Cloning	TRB1072/TRB1047	This work
pTRB491	pSAT1-LIC-*rv1044*	Ligation Independent Cloning	TRB1018/TRB1019	This work
pTRB493	pSAT1-LIC-*rv2827c*	Ligation Independent Cloning	TRB1022/TRB1023	This work
pTRB525	pSAT1-LIC-*abiEi*	Ligation Independent Cloning	TRB1048/TRB1049	This work

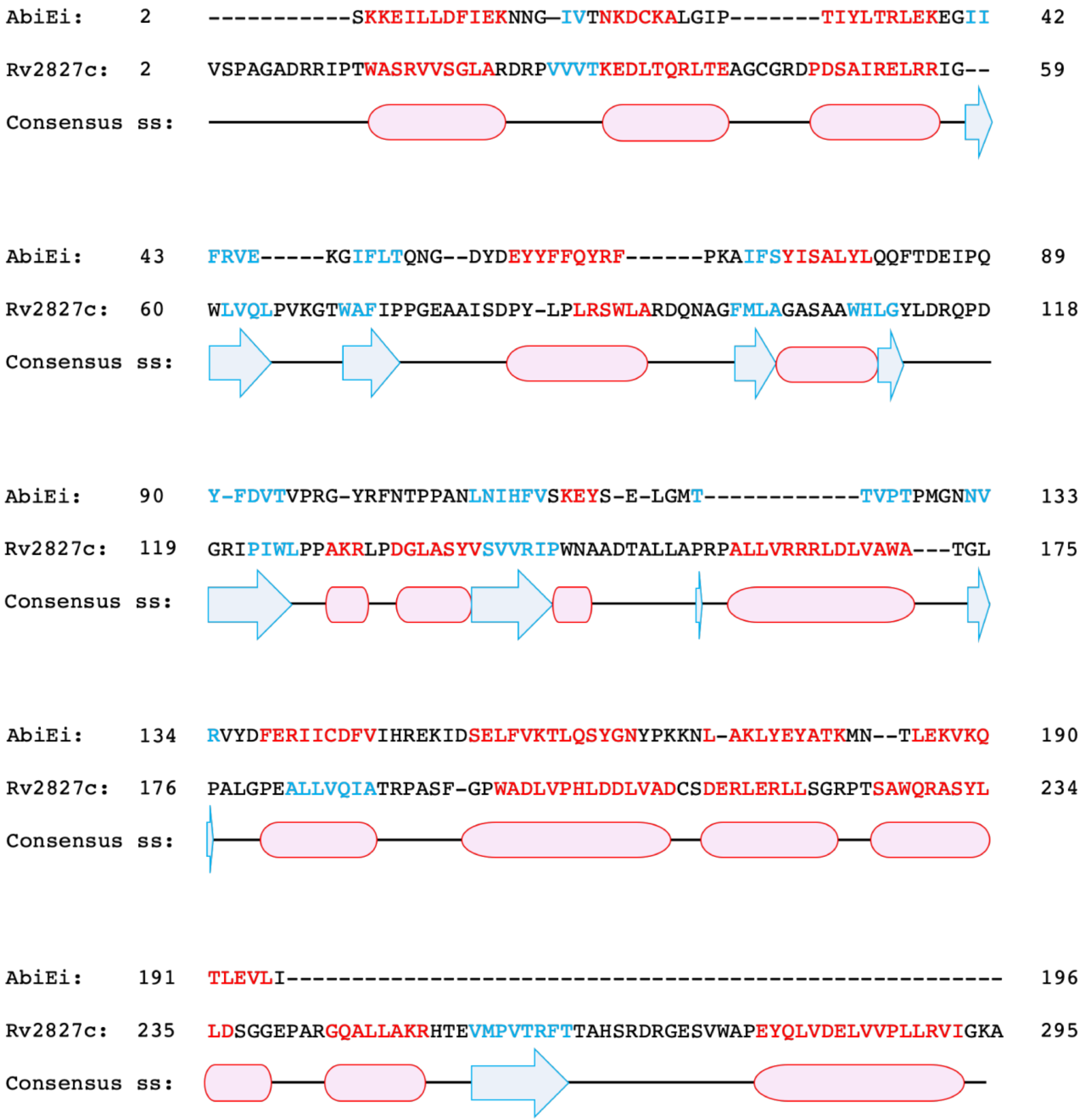
AbiEi: 2 -----------SKKEILLDFIEKNNG—IVTNKDCKALGIP-------TIYLTRLEKEGII 42
Rv2827c: 2 VSPAGADRRIPTWASRVVSGLARDRPVVVTKEDLTQRLTEAGCGRDPDSAIRELRRIG-- 59
Consensus ss:
AbiEi: 43 FRVE-----KGIFLTQNG--DYDEYYFFQYRF------PKAIFSYISALYLQQFTDEIPQ 89
Rv2827c: 60 WLVQLPVKGTWAFIPPGEAAISDPY-LPLRSWLARDQNAGFMLAGASAAWHLGYLDRQPD 118
Consensus ss:
AbiEi: 90 Y-FDVTVPRG-YRFNTPPANLNIHFVSKEYS-E-LGMT-----------TVPTPMGNNV 133
Rv2827c: 119 GRIPIWLPPAKRLPDGLASYVSVVRIPWNAADTALLAPRPALLVRRRLDLVAWA---TGL 175
Consensus ss:
AbiEi: 134 RVYDFERIICDFVIHREKIDSELFVKTLQSYGNYPKKNL-AKLYEYATKMN--TLEKVKQ 190
Rv2827c: 176 PALGPEALLVQIATRPASF-GPWADLVPHLDDLVADCSDERLERLLSGRPTSAWQRASYL 234
Consensus ss:
AbiEi: 191 TLEVLI-- 196
Rv2827c: 235 LDSGGEPARGQALLAKRHTEVMPVTRFTTAHSRDRGESVWAPEYQLVDELVVPLLRVIGKA 295
Consensus ss:

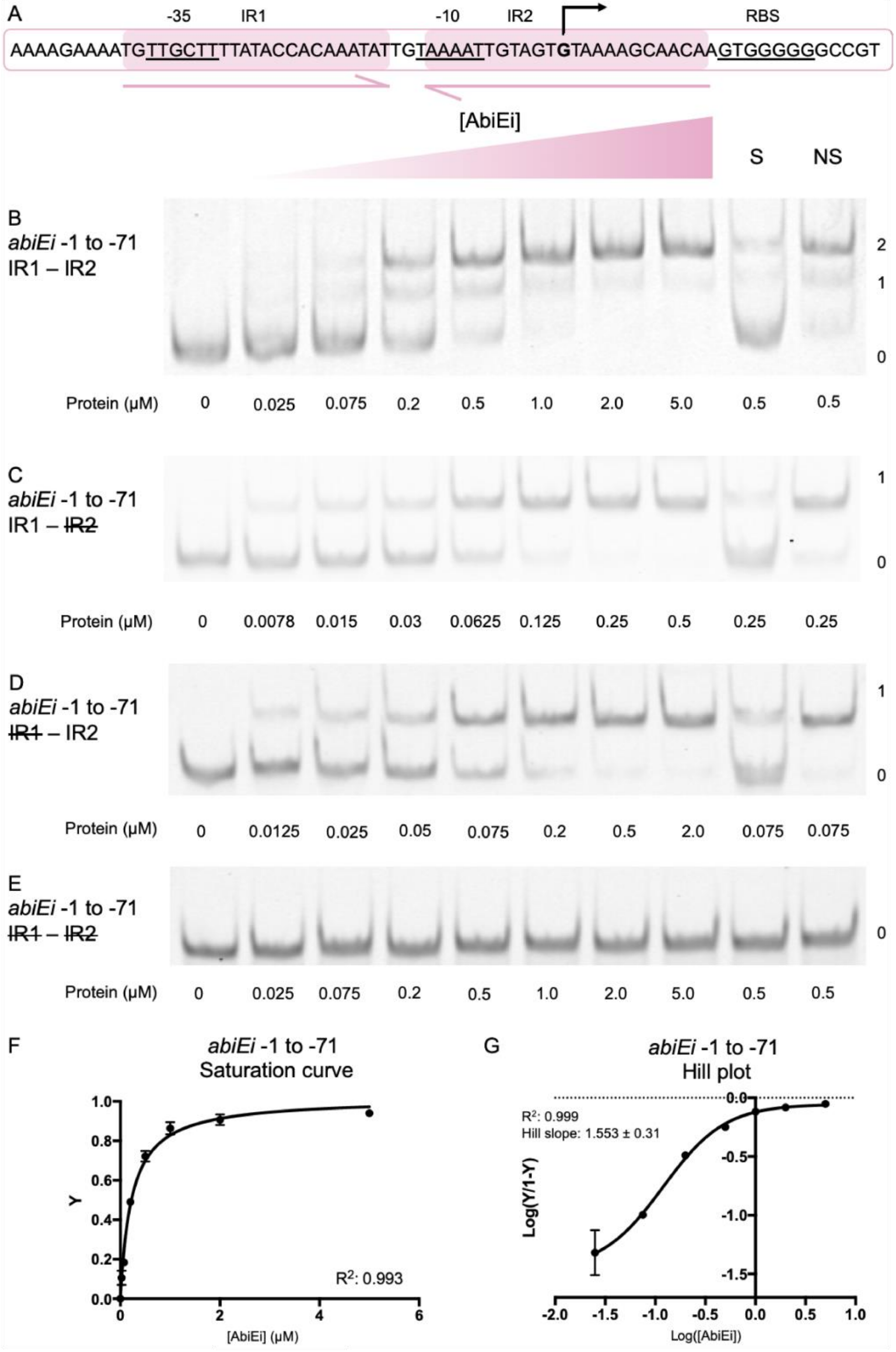

A
-35
IR1
-10
IR2
RBS
AAAAGAAAATGTTGCTTTATACCACAAATATTGTAAAATTGTAGTGTAAAAGCAACAAGTGGGGGCCGT
[AbiEi]
S
NS
B
abiEi -1 to -71
IR1 – IR2
2
1
0
Protein (μM) 0 0.025 0.075 0.2 0.5 1.0 2.0 5.0 0.5 0.5
C
abiEi -1 to -71
IR1 – IR2
1
0
Protein (μM) 0 0.0078 0.015 0.03 0.0625 0.125 0.25 0.5 0.25 0.25
D
abiEi -1 to -71
IR1 – IR2
1
0
Protein (μM) 0 0.0125 0.025 0.05 0.075 0.2 0.5 2.0 0.075 0.075
E
abiEi -1 to -71
IR1 – IR2
0
Protein (μM) 0 0.025 0.075 0.2 0.5 1.0 2.0 5.0 0.5 0.5
F
abiEi -1 to -71
Saturation curve
Y
R2: 0.993
[AbiEi] (μM)
G
abiEi -1 to -71
Hill plot
R2: 0.999
Hill slope: 1.553 ± 0.31
Log(Y/1-Y)
Log([AbiEi])

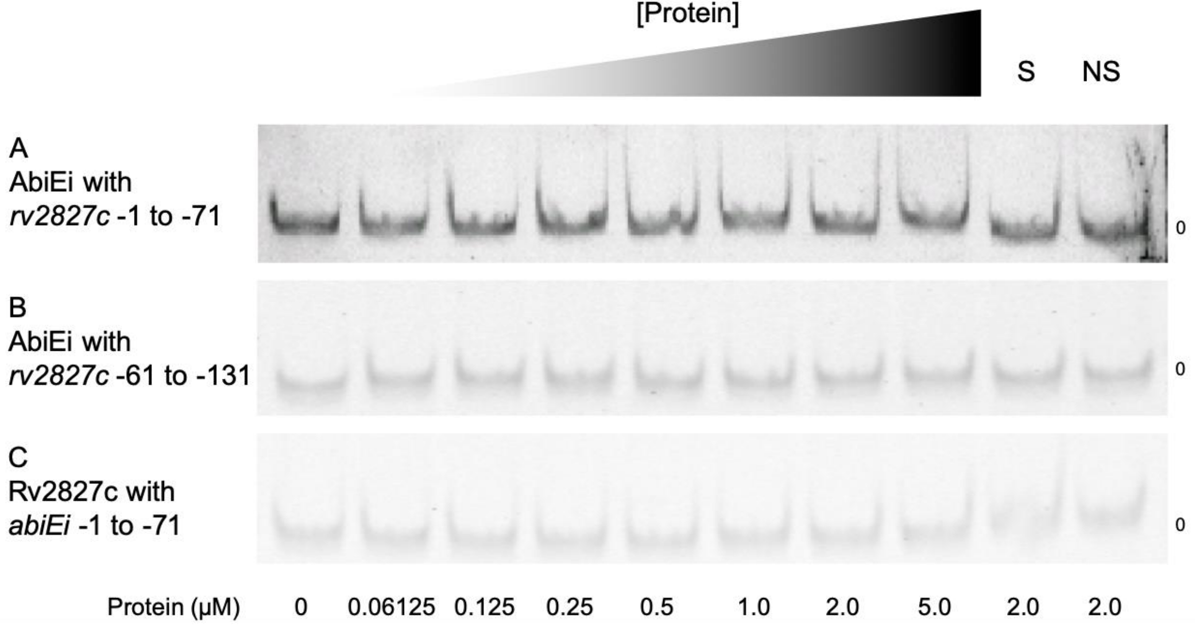
[Protein]
S
NS
A
AbiEi with
rv2827c -1 to -71
0
B
AbiEi with
rv2827c -61 to -131
0
C
Rv2827c with
abiEi -1 to -71
0
Protein (μM)
0
0.06125
0.125
0.25
0.5
1.0
2.0
5.0
2.0
2.0

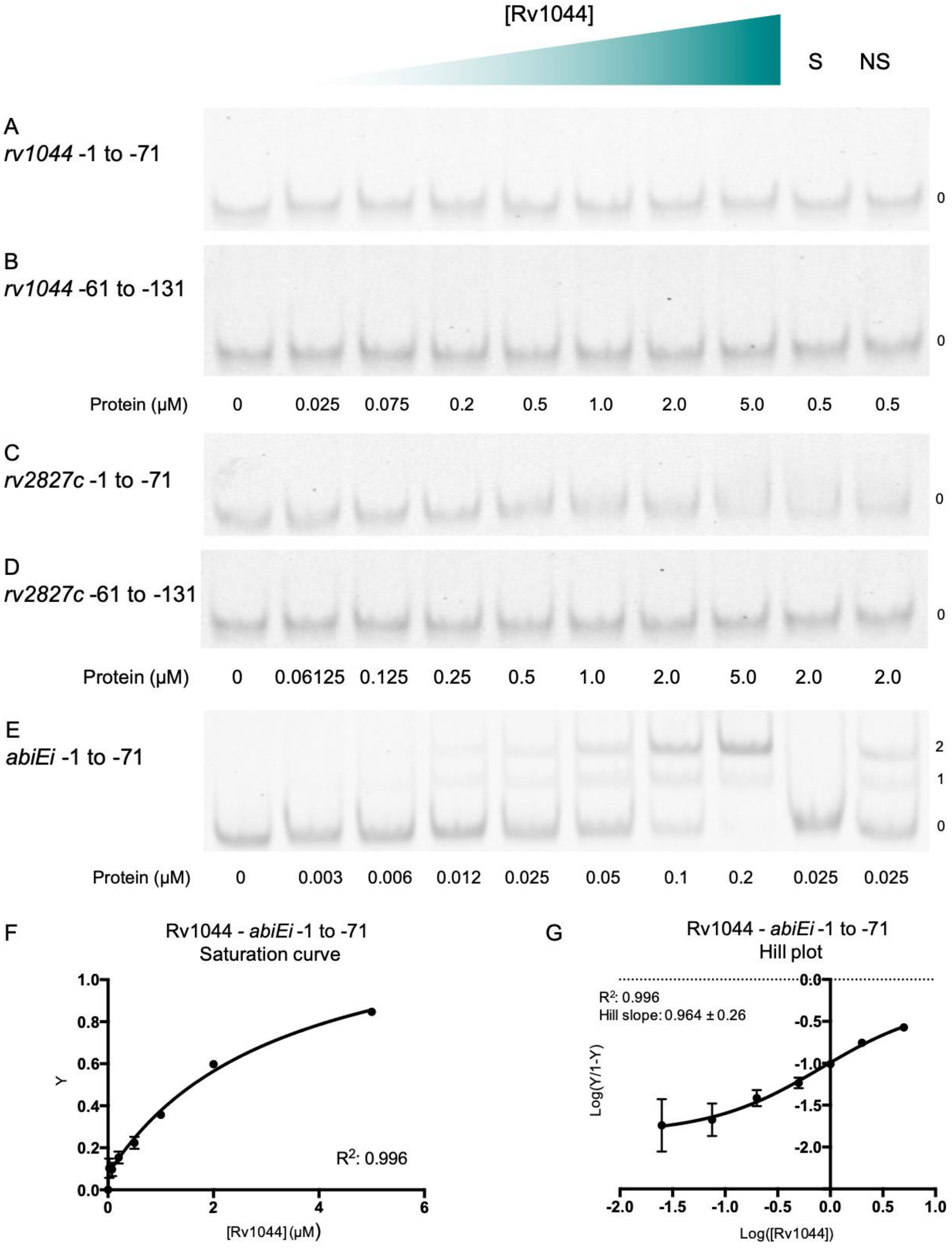

[Rv1044]
S
NS
A
rv1044 -1 to -71
0
B
rv1044 -61 to -131
0
Protein (μM) 0 0.025 0.075 0.2 0.5 1.0 2.0 5.0 0.5 0.5
C
rv2827c -1 to -71
0
D
rv2827c -61 to -131
0
Protein (μM) 0 0.06125 0.125 0.25 0.5 1.0 2.0 5.0 2.0 2.0
E
abiEi -1 to -71
2
1
0
Protein (μM) 0 0.003 0.006 0.012 0.025 0.05 0.1 0.2 0.025 0.025
F
Rv1044 - abiEi -1 to -71
Saturation curve
Y
[Rv1044] (μM)
R2: 0.996
G
Rv1044 - abiEi -1 to -71
Hill plot
R2: 0.996
Hill slope: 0.964 ± 0.26
Log(Y/1-Y)
Log([Rv1044])
